# Sequence and Structure Characteristics of 22 Deletion Breakpoints in Intron 44 of the *DMD* Gene Based on Long-Read Sequencing

**DOI:** 10.3389/fgene.2021.638220

**Published:** 2021-04-30

**Authors:** Chang Geng, Yuanren Tong, Siwen Zhang, Chao Ling, Xin Wu, Depeng Wang, Yi Dai

**Affiliations:** ^1^Department of Neurology, Peking Union Medical College Hospital, Chinese Academy of Medical Sciences, Beijing, China; ^2^GrandOmics Biosciences, Beijing, China; ^3^Laboratory of Clinical Genetics, Peking Union Medical College Hospital, Chinese Academy of Medical Sciences, Beijing, China

**Keywords:** Duchenne and Becker muscular dystrophy, *DMD* gene, copy number variations, long-read sequencing, NHEJ, MMEJ

## Abstract

**Purpose:** Exon deletions make up to 80% of mutations in the DMD gene, which cause Duchenne and Becker muscular dystrophy. Exon 45-55 regions were reported as deletion hotspots and intron 44 harbored more than 25% of deletion start points. We aimed to investigate the fine structures of breakpoints in intron 44 to find potential mechanisms of large deletions in intron 44.

**Methods:** Twenty-two dystrophinopathy patients whose deletion started in intron 44 were sequenced using long-read sequencing of a *DMD* gene capture panel. Sequence homology, palindromic sequences, and polypyrimidine sequences were searched at the breakpoint junctions. RepeatMasker was used to analyze repetitive elements and Mfold was applied to predict secondary DNA structure.

**Results:** With a designed *DMD* capture panel, 22 samples achieved 2.25 gigabases and 1.28 million reads on average. Average depth was 308× and 99.98% bases were covered at least 1×. The deletion breakpoints in intron 44 were scattered and no breakpoints clustered in any region less than 500 bp. A total of 72.7% of breakpoints located in distal 100 kb of intron 44 and more repetitive elements were found in this region. Microhomologies of 0–1 bp were found in 36.4% (8/22) of patients, which corresponded with non-homologous end-joining. Microhomologies of 2–20 bp were found in 59.1% (13/22) of patients, which corresponded with microhomology-mediated end-joining. Moreover, a 7 bp insertion was found in one patient, which might be evidence of aberrant replication origin firing. Palindromic sequences, polypyrimidine sequences, and small hairpin loops were found near several breakpoint junctions. No evidence of large hairpin loop formation in deletion root sequences was observed.

**Conclusion:** This study was the first to explore possible mechanisms underlying exon deletions starting from intron 44 of the *DMD* gene based on long-read sequencing. Diverse mechanisms might be associated with deletions in the *DMD* gene.

## Introduction

The *DMD* gene spans 2.3 Mb in chromosome X and is composed of 79 exons and lengthy introns (2.1 Mb) (Ahn and Kunkel, 1993). Mutations of *DMD* lead to progressive muscle weakness and degeneration, causing Duchenne muscular dystrophy (DMD; MIM:310200) and a milder phenotype, Becker muscular dystrophy (BMD; MIM:300376). Mutations on *DMD* can be divided into structural variations (SV) which include copy number variations (CNVs) and other complex rearrangements, and single nucleotide variations (SNVs). CNVs make up to 80% of the total mutations according to the global TREAT-DMD database (Bladen et al., 2015). Among the *DMD* mutation spectrum, deletions of one or more exons account for the majority, ranging from 43% to 80% in previous literature (Flanigan et al., 2009; Takeshima et al., 2010; Yang J. et al., 2013; Bladen et al., 2015; Tong et al., 2020). Based on intragenic analyses, the distribution of deletions was non-random, as notable deletion hotspots were found in central regions (exon 45-55) and 5′ regions (exon 2-20) (Bladen et al., 2015). Deletions which clustered in exon 45-55 took up to 60% of all deletion patterns (Flanigan et al., 2009; Takeshima et al., 2010; Yang J. et al., 2013; Bladen et al., 2015; Tong et al., 2020). Intron 44 spans the largest length (12%) of all introns in the *DMD* gene, while harboring more than 25% of the deletion start points (Tong et al., 2020). The percentage is far more out of proportion to its length, which may suggest that intron 44 may harbor specific sequence and structural features which are predisposed to large deletion.

High frequency of *de novo* mutations in *the DMD* gene has been observed, therefore, studies targeted at potential mechanisms are warranted. CNVs are generally believed to result from DNA replication-, recombination- and repair-associated mechanisms (Yang L. et al., 2013; Carvalho and Lupski, 2016). The main possible mechanisms known to cause CNV are non-allelic homologous recombination (NAHR), non-homologous end-joining (NHEJ), microhomology-mediated end-joining (MMEJ), and fork stalling and template switching (FoSTeS), which is also named microhomology-mediated break-induced replication (MMBIR). These different mechanisms can be inferred by different breakpoint sequence features (Kidd et al., 2010; Yang L. et al., 2013). Meanwhile, palindromic sequences, polypyrimidine sequences, and specific deletion related sequences as well as DNA secondary structures were also reported to mediate DNA deletion (Krawczak and Cooper, 1991; Trinh and Sinden, 1993; Rosche et al., 1995).

In previous research about mutations in the *DMD* gene, multiplex PCR, multiplex ligation dependent probe amplification (MLPA), array comparative genomic hybridization (aCGH), Sanger sequencing, and next-generation sequencing (NGS) were generally adapted. However, these techniques are either not accurate enough to provide the fine structures of breakpoints or too expensive and labor-intensive to detect mutations in large introns like intron 44, especially with a large sample volume. NGS were widely applied in recent research, yet it was insufficient for alignment in repetitive and complex regions like intron 44.

Previous studies, which relied on the above techniques about breakpoints in the *DMD* gene, suggested that intronic breakpoints of deletions were scattered across DMD and that there was no significant homology between proximal and distal breakpoints (Blonden et al., 1991; Love et al., 1991; Nobile et al., 2002; Toffolatti et al., 2002; Miyazaki et al., 2009; Esposito et al., 2017), which were consistent with NHEJ and MMEJ mechanisms. However, to our knowledge, although intron 44 is believed to harbor the majority of 5′ breakpoints of large deletions, few studies have focused on the sequence characteristics of deletion breakpoints in intron 44 (Blonden et al., 1991; Love et al., 1991; Miyazaki et al., 2009). Love et al. (1991) sequenced two patients and did not find any common DNA sequences adjacent to breakpoints except for AT-rich sequences. Blonden et al. (1991) mapped 242 patients’ DNA from intron 44-intron 45, relying on DNA hybridization techniques using whole cosmids as probes, and found no significant clustering of breakpoints. In another study, Japanese researchers mapped three patients with exon 45-55 deletions based on PCR and detected no substantial homologies across the breakpoints, which did not support homologous recombination mechanisms (Miyazaki et al., 2009). Not to mention that many studies only performed MLPA and did not search for breakpoints. Recently, Marey et al. (2016) mapped 39 patients with deletions starting from intron 44 based on an aCGH array, and found that 48.7% of breakpoints clustered in the distal 50 kb regions and 33.3% were clustered in regions less than 700 bp. Researchers also found that repetitive elements, and palindromic and T-A sequences were present in the vicinity of the breakpoints (Toffolatti et al., 2002; Marey et al., 2016).

In recent years, the rapidly developing third-generation single-molecule long-read sequencing technology has advanced in detecting genomic rearrangements, especially in repetitive or complex genomic regions, which may be problematic for NGS. With the long reads, researchers are able to discover genomic abnormalities as well as precisely find breakpoints at the base level. In order to precisely detect breakpoint sequences and evaluate secondary structures which may also contribute to genomic rearrangement, third-generation single-molecule long-read sequencing technology was used in a deletion involving intron 44 of the *DMD* gene in this research, so as to further investigate its potential mechanisms. Here, we performed whole *DMD* gene capture and long-read sequencing in 22 patients with large deletions whose breakpoints were located in intron 44. We aimed to explore sequence and structure characteristics of breakpoints in intron 44 so as to infer potential mechanisms of large deletions related to intron 44 in dystrophinopathy. As we know, this is the first study to use third-generation sequencing technology to explore deletions located in intron 44 of *DMD*.

## Materials and Methods

### Subjects

This research included 22 DMD/BMD patients carrying deletions with start breakpoints located in intron 44. Clinical data of the involved patients were extracted from the National Rare Diseases Registry System of China program (NRDRS-DMD/BMD database), which was described in detail before (Tong et al., 2020). All patients signed informed consents and this study was approved by the ethics committee of the Peking Union Medical College Hospital (IRB #JS-1233).

### Experiments

The reference sequence of the DMD gene was obtained from the University of California Santa Cruz (UCSC) human genome website^1^ based on human GRCh37/hg19. Customized DNA probes of 100 bases were designed to cover the DMD gene as well as its upstream and downstream 20 kb region (chrX:31115345-33377726). Probes corresponding to repetitive sequences in the human genome were excluded. Genomic DNA was extracted using the blood genome DNA extraction kit (Sangon Bioengineer Co., Shanghai, China) according to the manufacturer’s protocol. The DNA quantity and quality was assessed with Qubit 3.0 (Thermo Fisher Scientific Inc., Carlsbad, CA, United States) and agarose gel electrophoresis, respectively. A targeted sequencing library was prepared as following: 3 μg of genomic DNA per sample was sheared to 1 kb∼6 kb fragments by a g-Tube (#520079, Covaris) and centrifuged (1,5000 × *g*, 2 min, twice). The DNA was then purified after characterization of fragment size. End repair, A-tailing at the 3′ ends, and adapter ligation was performed through pre-capture amplification. Targeted sequence capture was conducted by pooling indexed PCR products and hybridization with custom capture probes. Purified DNA fragments were amplified by PCR and quantified, then they were subjected to sequencing on the long-read sequencing platform Oxford Nanopore PromethION with R9.4.1 flow cell according to the manufacturer’s standard protocols.

### Sequencing Data Analysis

ONT official basecaller Guppy (version 3.0.5 + 45c3543) was invoked in basecalling from FAST5 format raw sequencing data to FASTQ files with high accuracy mode. Reads with quality >7 were kept and demultiplexed into separated samples by nanoplexer (version 0.1) (Han, 2020). Each sample was mapped against reference genome (hg19, UCSC) by minimap2 (version 2.15-r906-dirty) (Heng, 2018), and then SV detection was performed through Sniffles (version 1.0.11) (Sedlazeck et al., 2018). In order to generate breakpoint consensus sequences, reads spanning the breakpoints were extracted and self-aligned by spoa (version v3.0.1) (Vaser et al., 2017) which implemented the partial order alignment (POA) algorithm. The consensus sequences were aligned to a reference using minimap2 to inspect small indels of breakpoints.

The RepeatMasker program^2^ was used for searching interspersed repeats and low complexity DNA sequences with default settings, for example, long interspersed nuclear elements (LINE) and short interspersed nuclear elements (SINE) including Alu elements and long terminal repeat (LTR) elements. The breakpoint junction sequences of 22 patients were confirmed and located by the BLAT program in the UCSC human genome website^3^ Palindromic sequences (≥6 bp), polypyrimidine sequences (≥7 bp), and specific sequences of TTTAAA and TG(A/G)(A/G)(G/T)(A/C) were searched for in the breakpoint junction sequences. Secondary structures including small hairpin structures were predicted and analyzed based on the Mfold program following the lowest energy principle (Sedlazeck et al., 2018)^4^.

Longest common substring (LCS), longest common subsequence (LCSeq), Levenshtein distance, and Hamming distance were used as indicators of similarity (Dinu and Sgarro, 2006; Alsmadi and Nuser, 2012). Patients’ and the corresponding reference intron sequences near the breakpoint were extracted and the four indicators of similarity were calculated. Besides, similarity of the root segments of potential hairpin structures (i.e., downstream sequences of start breakpoints and upstream sequences of end breakpoints) were also analyzed. Sequences of root segments were extracted from reference DMD sequences and one of the two extracted sequences was conversed to its complementary sequence. We randomly generated 10,000 DNA sequences and calculated the same indicators as control groups.

Python 3.6 was utilized for searching for deletion-related sequences among breakpoint junction sequences and similarity analyses. Statistical analyses were performed by R software version 4.0.0, and ggplot2 was invoked in plotting.

## Results

### Sequencing Data Profiles

**FIGURE 1:**
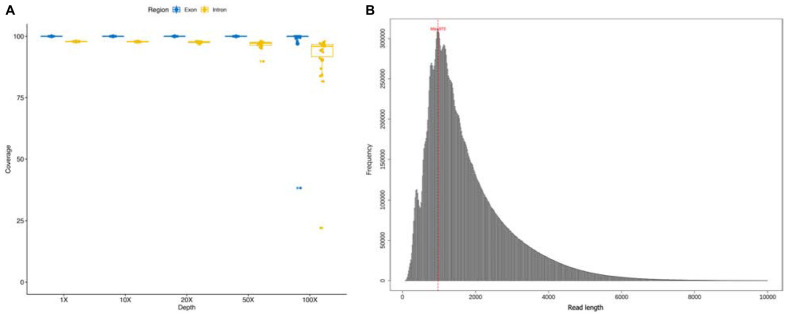
**(A)** Coverage ratio in different depth level of each sample; **(B)** the length of sequencing reads of each sample.

A total of 22 DMD patients with deletions starting from intron 44 were involved in this study, and sequenced using a customized whole *DMD* gene capture panel. The average read number and mean base number of the 22 enrolled samples were 1.28 million and 2.25 gigabases, correspondingly. An average mapping ratio as high as 98.07% was achieved with a standard deviation of 0.23%. Mean target reads ratio and target average depth of all samples were 36.90% and 308×, ranging from 19.11% to 48.93% and 75× to 681×, respectively. On average, 22 samples covered 2,261,985 bases of the target region, accounting for a coverage ratio of 99.98%. For each sample, the coverage ratio in different depth levels was calculated to ensure the absence of capture and sequence bias, shown in Figure 1A. The deleted genomic regions were excluded in the summarization. Moreover, the length of sequencing reads of all samples were distributed in a histogram with 20 bp size in Figure 1B, reads whose length was greater than 10 kb were excluded in the figure. The mean length of sequencing reads was 1.8 kb and median length was 1.45 kb. The sequencing statistics of the 22 samples are summarized in Supplementary Table 1. Across 79 exons and 78 introns of the *DMD* gene, the average depth of each genomic functional area resembled each other, for details see Supplementary Table 2.

### Intron 44 Structure Characteristics

Intron 44 had a length of 248 kb, accounting for 12% of the total length of the DMD gene. Compared with the *DMD* gene, intron 44 had a higher proportion of special elements, including mammalian-wide interspersed repeats (MIR) (3.26% vs. 2.70%), long interspersed elements 2 (LINE2) (6.88% vs. 4.55%), and hAT-Charlie elements (3.07% vs. 2.18%) (see Table 1).

**TABLE 1:** Repetitive sequences in the *DMD* gene and intron 44 of the *DMD* gene.

		**DMD**	**Intron 44**	**Distal 100 kb of intron 44**
				**31986632-32086631**
Total length (bp)		2262381	248401	100000
GC level		36.42%	36.17%	36.84%
**Sequence types (length occupied)**				
SINEs:		8.83%	7.14%	8.93%
	ALUs	6.10%	3.87%	5.39%
	MIRs	2.70%	3.26%	3.54%
LINEs:		19.32%	15.45%	12.51%
	LINE1	14.00%	7.76%	4.24%
	LINE2	4.55%	6.88%	7.43%
	L3/CR1	0.59%	0.60%	0.40%
LTR elements:		7.06%	5.44%	7.37%
	ERVL	1.77%	1.40%	1.09%
	ERVL-MaLRs	2.99%	2.71%	4.16%
	ERV_class I	2.12%	1.23%	2.12%
	ERV_class II	0.00%	0.00%	0.00%
DNA elements:		4.45%	4.13%	5.54%
	hAT-Charlie	2.18%	3.07%	4.26%
	TcMar-Tigger	1.40%	0.41%	0.84%
Total interspersed repeats:		39.78%	32.43%	34.87%
Simple repeats:		1.33%	1.23%	1.35%
Low complexity:		0.20%	0.11%	0.09%

*DMD, Duchenne muscular dystrophy; SINEs, short interspersed elements; MIRs, mammalian-wide interspersed repeats; LINEs, long interspersed elements; LTR, long terminal repeat elements; DNA elements, DNA repeat elements.*

Further comparison was conducted between distal 100 kb and whole intron 44. The distal 100 kb at the 3′ direction of intron 44 had a higher proportion of Alu family (5.39% vs. 3.87%), MIR (3.54% vs. 3.26%), and LINE2 (7.43% vs. 6.88%) (see Table 1).

### Breakpoint Distribution and Structure Characteristics

**FIGURE 2:**
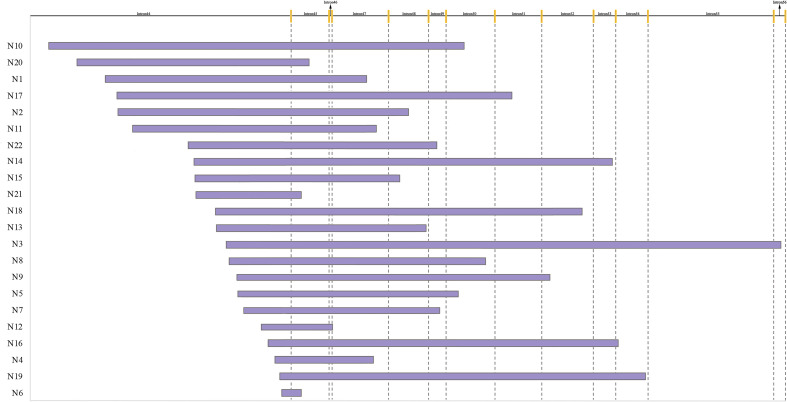
Start and end points of 22 patients with deletion. The yellow line indicates different exons.

As showed in Figure 2, the distribution range of breakpoints covered 89.1% of the total intron 44, while deletion start points of 16 (72.7%) patients were in the region of the distal 100 kb of intron 44 and end points distributed from intron 45 to intron 56. However, no breakpoints clustered in any region which was less than 500 bp. The lengths of deleted base pairs ranged from 19 kb to 530 kb and the average deletion length was 253 kb. The distribution of breakpoints as well as special elements in intron 44 are presented in Supplementary Figure 1.

A total of 36.4% (8/22) of patients’ deletion mutations started from special elements, while 18.2% (4/22) of them were located in LINE (Table 2 and Supplementary Table 3). There were no significant differences in the distribution of breakpoints in special elements in intron 44 and other introns. Most patients had microhomologies between sequences near the start and end points: 36.4% (8/22) of patients had 0–1 bp microhomologies, while 59.1% (13/22) of patients had 2–20 bp microhomologies. A 7 bp insertion was found in one patient. Low-copy repeats (LCR) were not found in all patients (Table 3).

**TABLE 2:** Special elements at breakpoints in intron 44 and other introns.

**Elements**	**Breakpoints in intron 44**	**Breakpoints in other introns**	***P*-value**
Unique	14 (63.7%)	11 (50.0%)	0.54
**Special sequence**			0.75
LINE	4 (18.3%)	4 (18.3%)	
SINE	1 (4.5%)	1 (4.5%)	
DNA	1(4.5%)	3 (13.6%)	
LTR	1(4.5%)	3 (13.6%)	
Low complexity	1(4.5%)	0 (0.0%)	

*LINEs, long interspersed elements; SINEs, short interspersed elements; DNA elements, DNA repeat elements; LTR, long terminal repeat elements.*

**TABLE 3:** Breakpoint structure characteristics and potential mechanisms.

**Breakpoint structure characteristic**	**Number**	**Percentage (%)**	**Potential mechanism**
**No additional sequence at breakpoint**			
0 or 1 matching nucleotides	8	36.4%	NHEJ
2–20 matching nucleotides	13	59.1%	MMEJ
>20 matching nucleotides	0	0.0%	
**Additional sequence at breakpoint**			
1–10 additional nucleotides	1	4.5%	AROR
>10 additional nucleotides	0	0.0%	

*NAHR, non-allelic homologous recombination; NHEJ, non-homologous end-joining; MMEJ, microhomology-mediated end-joining; AROR, aberrant replication origin firing.*

**FIGURE 3:**
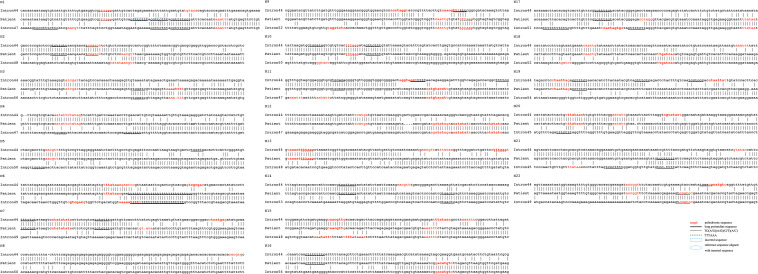
Genomic sequences spanning 22 deletion breakpoints with corresponding reference intron sequences. Only the 5′–3′ strands are shown. Palindromic sequences are presented in red color. Black underlines indicate long pyrimidine sequences, black dotted lines indicate TG(A/G)(A/G)(G/T)(A/C), and red dotted lines indicate TTTAAA. The inserted sequence is highlighted in a blue rectangle and the reference sequence that was aligned with the inserted sequence is highlighted in a blue circle.

**FIGURE 4:**
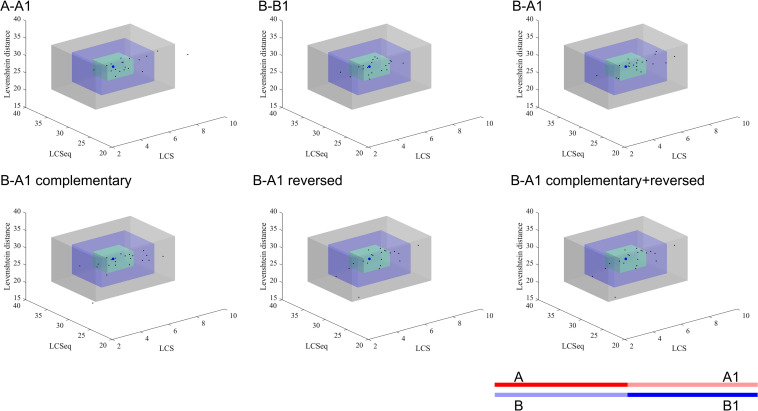
Similarity analyses of subsequences near the breakpoints. The blue dot represents the mean value of the random sequences and each black dot presents the index data of a patient. The green, blue, and gray cuboid indicates 1, 2, and 3 standard deviations from the mean value, respectively. A, A1, B, and B1 represent different segments of the corresponding gene sequences as the legend shows. A: upstream 50 bp of start point; A1: downstream 50 bp of start point of reference sequence; B: upstream 50 bp of end point of reference sequence; B1: downstream 50 bp of end point. The semitransparent lines show the deleted sequences.

Figure 3 shows the special sequences of all patients, including palindromic sequences (≥6 bp), polypyrimidine sequences (≥7 bp), and TG(A/G)(A/G)(G/T)(A/C) sequences. The median number of palindromic sequences in the surrounding 100 bp region of each breakpoint junction was 2 (range: 0–5). Polypyrimidine sequences in the vicinity of breakpoint junctions were found in 45.5% (10/22) of patients, while four patients had TTTAAA and five patients had TG(A/G)(A/G)(G/T)(A/C) sequences. Special sequences in reference introns are also marked in Figure 3. Similarity analyses of subsequences near the breakpoints are displayed in Figure 4. No overall significant differences were found when comparing LCS, Levenshtein distance, and Hamming distance between target sequences with random sequences.

**FIGURE 5:**
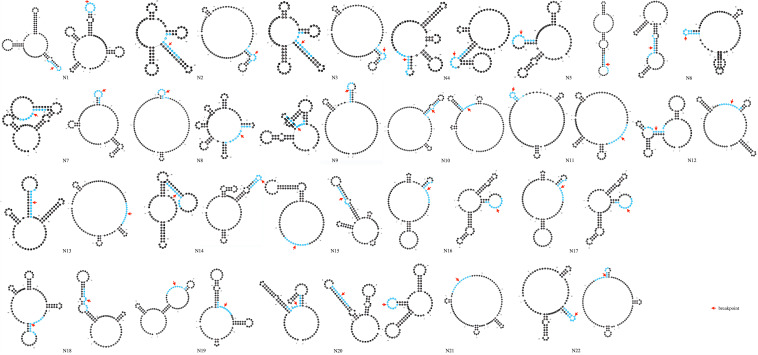
Prediction of secondary structure of 100 bp spanning deletion start and end points of 22 patients. Red arrow indicates the breakpoint. 10 bp around the breakpoint is highlighted in blue color.

Secondary structure analysis showed that 75.0% (33/44) of breakpoints were located in hairpin loops and 36.4% (16/44) of breakpoints were located in the non-matching section of the hairpin structure, as shown in Figure 5.

The similarity analyses of the root area of potential hairpin structures are shown in Table 4. No overall significant differences were found when comparing LCS, Levenshtein distance, and Hamming distance between target sequences with random sequences.

**TABLE 4:** Similarity analyses of the root area of potential hairpin structure.

	**LCS**				**LCSeq**				**Levenshtein distance**				**Hamming distance**			
	**10 bp**	**20 bp**	**30 bp**	**100 bp**	**10 bp**	**20 bp**	**30 bp**	**100 bp**	**10 bp**	**20 bp**	**30 bp**	**100 bp**	**10 bp**	**20 bp**	**30 bp**	**100 bp**
D2	2	6	7	7	4	10	18	64	7	13	16	52	7	15	18	63
D6	5	5	5	6	7	13	20	59	5	9	14	60	7	13	20	64
D7	2	5	5	8	5	10	18	66	6	12	16	50	6	12	21	68
D8	4	5	5	6	5	10	17	64	7	14	19	53	7	15	21	72
D9	2	4	5	5	6	11	19	62	7	13	19	57	8	16	25	74
D10	3	3	4	6	7	9	15	55	3*	11	17	60	3	11	19	76
D11	4	5	5	6	6	10	16	63	6	12	18	53	8	17	25	78
D14	3	4	4	8	4	10	15	60	7	12	19	57	9	16	24	81
D16	3	4	4	5	5	10	16	61	7	14	21	57	7	17	26	86
D17	3	4	5	5	5	11	16	63	7	13	19	55	8	15	21	65
D21	2	3	3	5	3	7#	11	55#	8	16	22	59	8	16	22	72
D22	3	3	5	7	5	13	20	66	7	10	14	50	8	15	22	70
D25	3	4	4	6	5	11	17	65	6	12	17	54	9	17	23	66
D28	2	3	3	5	5	10	17	61	6	14	19	56	6	15	21	77
D29	2	4	5	6	4	11	17	60	8	12	18	57	9	16	24	76
D31	4	6	7	9*	6	14	21	63	5	10	15	53	6	13	18	69
D32	4	4	5	6	7	15	19	65	4	9	15	49	4	9*	17	69
D35	4	4	4	8	5	11	17	64	6	12	17	53	8	17	24	79
D38	3	3	4	7	4	9	15	64	6	13	18	56	6	13	20	74
D49	2	3	6	6	5	12	17	64	8	12	18	55	8	17	25	75
D50	3	7*	7	7	7	12	19	60	4	12	17	56	5	15	21	74
D51	4	5	5	6	6	11	18	56	6	12	17	58	6	14	23	75
Mean	3.05	4.27	4.86	6.36	5.27	10.91	17.18	61.82	6.18	12.14	17.50	55.00	6.95	14.73	21.82	72.86
SD	0.88	1.09	1.10	1.11	1.09	1.73	2.12	3.23	1.30	1.63	1.99	3.06	1.55	2.09	2.48	5.64
Reference	2.74 ± 0.84	3.79 ± 0.92	4.47 ± 0.94	6.22 ± 0.90	5.26 ± 1.03	11.41 ± 1.26	17.61 ± 1.40	62.16 ± 2.13	6.56 ± 1.18	12.28 ± 1.55	17.87 ± 1.75	55.78 ± 2.65	7.54 ± 1.40	15.02 ± 1.97	22.63 ± 2.48	75.13 ± 4.15
*P*-value	0.13	0.06	0.12	0.56	0.96	0.20	0.37	0.63	0.20	0.69	0.41	0.26	0.10	0.53	0.15	0.08

*The value was beyond (mean + 3*SD) of the reference sequence for LCS and LCSeq or (mean – 3*SD) of the reference sequence for Levenshtein distance and Hamming sequence, which was considered as unusual high similarity. ^#^The value was beyond (mean – 3*SD) of the reference sequence for LCS and LCSeq or (mean + 3*SD) of the reference sequence for Levenshtein distance and Hamming sequence, which was considered as unusual low similarity. *P* < 0.05 was considered as a statistically significant difference in similarity between test sequences and references. LCS, longest common substring; LCSeq, longest common subsequence; SD, standard deviation.

## Discussion

Our study provides sequence analyses of the genomic architecture of intron 44 and structural characterization of 22 breakpoint junctions in patients with deletions starting from intron 44 based on third-generation long-read sequencing, leading to proposals of potential mechanisms of large deletion of DMD.

Intron 44 spans the largest length (12%) of all introns in the *DMD* gene, and harbors more than 25% of the deletion start points (Tong et al., 2020). However, only a few previous small-sample studies have focused on the fine structure of intron 44 (Blonden et al., 1991; Love et al., 1991; Miyazaki et al., 2009; Marey et al., 2016). The main difficulty for the sequence analysis of intron 44 may be that short-read sequencing is not adept at locating the exact breakpoints in such a large intron with tremendous repetitive regions. Some NGS studies applied coverage depth, read-pair, and split-read to detect deletions and duplications (Lim et al., 2011; Wei et al., 2014). But the resolution was only exon-level, similar to MLPA. And the coverage depth could be influenced by capture assay and sequencing parameters. For example, Gonçalves et al. (2017) reported a case that an exonized LINE-1 deeply inserted in intron 51 was genotyped only by RNA-sequencing and a single-molecule sequencing technique, but routine MLPA and NGS failed to detect it. However, the exact sites of breakpoints do have great value in clinical and research fields. Greer reported a patient harboring a deletion in *DMD* between exon 45 and exon 47 presenting an obvious more severe phenotype than BMD. The reason was a 82-bp pseudoexon derived from the novel sequence around the junction of intron 44 and intron 47 inserted into the dystrophin mRNA (Greer et al., 2015).

In the era of gene therapy, the information of the exact boundaries of large deletion and duplication is more significant than before. Array CGH used probes that covered both dystrophin exons and introns, which allowed for pinpointing locations of both exonic and intronic breakpoints. However, the resolution of CGH was at the hundred-base-pairs level, thus fine structures of breakpoints could not be obtained. NGS could obtain the whole *DMD* gene sequence, but the capture, alignment, and mapping processes by short-read sequencing in large and complex intronic regions are not satisfying. Sanger sequencing could identify an intronic sequence step by step, but it is labor-intensive and costly and could not be applied routinely.

In recent decades, the development of third-generation long-read sequencing has enabled researchers to align repeat-rich regions with sufficient anchors within the flank. Long-read sequencing allows for the discovery of genomic abnormalities as well as the location of breakpoints at the base pair level. Therefore, it is possible to genotype the fine structure of CNVs and complex genomic rearrangements with high confidence based on third-generation sequencing (Huddleston et al., 2017; De Coster et al., 2018; Mantere et al., 2019). So far to our knowledge, our study is the first to apply third-generation sequencing to explore the genomic landscape in the largest intron in *DMD*. Compared with previous methods, long-read sequencing could achieve a high average mapping ratio, nearly 100% coverage for exons and introns, and a satisfying target reads ratio and target average depth, whose standard deviations were relatively low. The average depth of genetic functional regions such as exons, introns, and untranslated regions were relatively similar, indicating low-rate absence of capture and sequencing bias.

Non-random distribution of breakpoints in intron 44 was observed in our study, with 72.7% (16/22) clustered in the distal 100 kb region, which was consistent with previous studies. Miyazaki et al. (2009) reported that two breakpoints of three patients were distributed in the 3′ part in intron 44. Marey et al. (2016) also observed that 48.7% (19/39) of breakpoints clustered within the distal 50 kb region of the intron. Therefore, based on the results of our study and previous research, we inferred that this specific genomic landscape may predispose the 3′ region of intron 44 to more CNVs.

The main mechanisms underlying CNVs included NAHR, NHEJ, MMEJ, FoSTeS/MMBIR, and others. Recurrent recombination and unequal cross-over between two lengths of DNA with high similarity leads to NAHR, which is usually responsible for recurrent CNVs whose breakpoints cluster in homologous low-copy repeats, for example, the pathogenesis of Charcot-Marie-Tooth disease type 1A and hereditary neuropathy with liability to pressure palsies. As shown in Figure 2, the deleted segments were totally different and the breakpoints were scattered over a large range. In addition, no extensive homology was found at the junctions in the similarity analyses in our study. No evidence supports NAHR mechanisms causing deletions in the *DMD* gene. So along with previous studies, we suggested that NAHR is not a main mechanism in CNV formation of dystrophinopathy.

Genomic arrangements could also arise from repair pathways of double-strand DNA breakage (DSB) without requiring extensive homology like NAHR, for example, NHEJ and MMEJ. NHEJ is usually accompanied with 0–1 bp matching nucleotides and short insertions while 2–20 bp matching nucleotides can be found in MMEJ (Kidd et al., 2010). Our study demonstrated that all breakpoints were scattered with no breakpoints clustering in any 500-bp region, which indicated the somewhat random nature in the occurrence of large deletions. Besides, non-homology and microhomology were dominant at the junctions (36.4% for 0–1 matching nucleotide, 4.5% for short insertions, and 59.1% for 2–20 matching nucleotides), suggesting that most deletions were generated via mechanisms mediated by NHEJ and MMEJ. Previous studies which focused on other introns in the *DMD* gene also found that NHEJ or MMEJ play important roles in large deletions in the *DMD* gene (Blonden et al., 1991; Love et al., 1991; Nobile et al., 2002; Toffolatti et al., 2002; Miyazaki et al., 2009; Esposito et al., 2017), which was consistent with our study. For example, Blonden et al. (1991) and Love et al. (1991) found no significant clustering of breakpoints and no common sequences adjacent to breakpoints. In addition, Miyazaki et al. (2009) detected no substantial homologies across the breakpoints in three patients with exon 45-55 deletions, which did not support homologous recombination mechanisms.

Intron 44 was associated with a high ratio of MIR, LINE2, and hAT-Charlie elements compared with the whole *DMD* gene as reference, which may indicate that NHEJ/MMEJ are likely mediated by repetitive elements. Furthermore, a high ratio of the same repetitive elements as well as the Alu element was also found in the distal 100 kb of intron 44 compared with the whole intron. Combined with the distribution features of breakpoints and previous literature, we suggested that the relatively high ratio of repetitive elements in intron 44 (mostly in the distal region) may predispose intron 44 to DSB and repairment mismatch through long-range effects, leading to a large number of deletion start points in the vicinity of repetitive elements (Shaw and Lupski, 2004).

Other replication-based models were also proposed to explain complex genetic rearrangement including FoSTeS/MMBIR and AROR. In the MMBIR model, the replication fork can stall and switch templates via microhomology to other sequences and often lead to inversion, translocation, or more complexed rearrangements (Hastings et al., 2009; Zhang et al., 2009). But in our study, we did not observe similar rearrangements that may be caused by MMBIR. In addition, Ankala et al. (2012) hypothesized that AROR – aberrant firing of replication origins with tandem repetitions of short reference sequences – were proximal to the breakpoints. In our study, the D2 patient also had a 7 bp insertion. The inserted sequence was aligned with the reference sequence proximal to the breakpoint, which may serve as evidence of the replication slippage and re-replication theory.

Moreover, factors that may interrupt the replication process thus leading to deletion formation were observed in intron 44. We observed that four breakpoint junctions were accompanied with the TTTAAA sequence, which was related to the curvature of DNA. And the frequency was higher than what was expected based on the frequency in the human genome (1/1420 bp) (Drmanac et al., 1986). Palindromic sequences were common in the surrounding 100 bp of breakpoint junctions, which may also lead to the formation of small hairpin loops thus contributing to the occurrence of large deletions. In conclusion, the results were consistent with previous research. Love et al. (1991) found AT-rich sequences adjacent to breakpoints. Toffolatti et al. (2002) and Marey et al. (2016) found that repetitive elements, and palindromic and T-A sequences were present in the vicinity of the breakpoints. Besides, hairpin loops in a single strand of DNA are associated with genetic instability (Sheng et al., 2003). In our study, most of the deletion breakpoints (75.0%) were located in hairpin loops and 36.4% of breakpoints were within an unmatched area of the hairpin structures.

We also performed similarity analyses between nucleotides at the start and end regions of deleted sequences, in order to test the hypothesis whether a deletion was caused when a huge hairpin loop structure was formed. The LCS is the length of the longest common consecutive substring, which focuses on local similarities, emphasizing complete complementary short sequences. The Hamming distance between two strings equals the number of positions at which the corresponding symbols are different, which measures the minimum substitutions required to transform one string to the other. Hamming distance is a trivial index which could show similarities directly. The Levenshtein distance between two strings is defined as the number of insertions, deletions, and substitutions that are required to transform from one string to the other. Levenshtein distance compares the two sequences in general. Combining these three indexes together, we were able to observe the similarity result comprehensively. All indexes indicated that neighbor areas of breakpoints of deleted sequences did not show higher similarities than random sequences. Therefore, the possibility for large hairpin loop formation in deletion sequences is low. In addition, though no statistically higher similarity was found in the upstream/downstream sequences of start and end points compared to the reference sequence, we still observed a trend that A-A1 and B-B1 tended to have longer LCSeq and LCS, based on which we inferred that deletion may prefer to happen between two similar DNA fragments to some extent.

There are some limitations of our study. First, the limited number of enrolled patients may reduce the significance of our study. More patients are expected to be enrolled in the future. Still, our study recruited the largest samples of deletions beginning at intron 44 compared with previous studies. In addition, different mechanisms require different operating proteins, for example, Ku70 and XRCC4 are necessary in NHEJ while Rad51 is required in NAHR (Shaw and Lupski, 2004; Chaplin and Blundell, 2020). Analysis of protein recruitment will provide further evidence of potential mechanisms.

In conclusion, this work was the first study to explore possible mechanisms underlying large deletions starting from intron 44 of the *DMD* gene based on third-generation long-read sequencing. Based on our results, diverse mechanisms are associated with CNVs in the *DMD* gene. Repetitive elements, palindrome sequences, short tandem repeats, and small hairpin loops may lead to genetic instability. When DSBs happened, NHEJ and MMEJ were enabled and then CNVs took place. Other mechanisms may also be related with deletion formation. Our study provides insights into the molecular pathogenesis of dystrophinopathy and indicates novel explanations for phenotypic differences between patients with the same exonic deletions.

## Data Availability Statement

The data presented in the study are deposited in the Genome Sequence Archive for Human, under accession number HDAC000390.

## Ethics Statement

The studies involving human participants were reviewed and approved by the Ethics Committee of the Peking Union Medical College Hospital. Written informed consent to participate in this study was provided by the participants’ legal guardian/next of kin.

## Author Contributions

YD, CG, and YT designed the study. SZ and DW collected and analyzed the data. CG, YT, and SZ performed all analyses and wrote the draft. CL, XW, and YD revised the manuscript. All authors have approved the manuscript to be published.

## Conflict of Interest

SZ, XW, and DW are employees of GrandOmics Biosciences. The remaining authors declare that the research was conducted in the absence of any commercial or financial relationships that could be construed as a potential conflict of interest.

## Footnotes

1 http://genome.ucsc.edu

2 http://www.repeatmasker.org

3 http://genome.ucsc.edu/cgi-bin/hgBlat

4 http://unafold.rna.albany.edu/?q=mfold/DNA-Folding-Form

## References

[B1] Ahn A. H. Kunkel L. M. (1993). The structural and functional diversity of dystrophin. *Nat. Genet.* 3 283–291. 10.1038/ng0493-283 7981747

[B2] Alsmadi I. Nuser M. (2012). String matching evaluation methods for DNA comparison. *Int. J. Adv. Sci. Tech.* 47 13–32.

[B3] Ankala A. Kohn J. N. Hegde A. Meka A. Ephrem C. L. H. Askree S. H. (2012). Aberrant firing of replication origins potentially explains intragenic nonrecurrent rearrangements within genes, including the human DMD gene. *Genome. Res.* 22 25–34. 10.1101/gr.123463.111 22090376 PMC3246204

[B4] Bladen C. L. Salgado D. Monges S. Foncuberta M. E. Kekou K. Kosma K. (2015). The TREAT-NMD DMD Global Database: analysis of more than 7,000 Duchenne muscular dystrophy mutations. *Hum. Mutat.* 36 395–402. 10.1002/humu.22758 25604253 PMC4405042

[B5] Blonden L. A. Grootscholten P. M. den Dunnen J. T. Bakker E. Abbs S. Bobrow M. (1991). 242 breakpoints in the 200-kb deletion-prone P20 region of the DMD gene are widely spread. *Genomics* 10 631–639. 10.1016/0888-7543(91)90445-K 1679746

[B6] Carvalho C. M. Lupski J. R. (2016). Mechanisms underlying structural variant formation in genomic disorders. *Nat.* R*ev. Genet.* 17 224–238. 10.1038/nrg.2015.25 26924765 PMC4827625

[B7] Chaplin A. K. Blundell T. L. (2020). Structural biology of multicomponent assemblies in DNA double-strand-break repair through non-homologous end joining. *Curr. Opin. Struct. Biol.* 61 9–16. 10.1016/j.sbi.2019.09.008 31733599

[B8] De Coster W. D’Hert S. Schultz D. T. Cruts M. Van Broeckhoven C. (2018). NanoPack: visualizing and processing long-read sequencing data. *Bioinformatics* 34 2666–2669. 10.1093/bioinformatics/bty149 29547981 PMC6061794

[B9] Dinu L. I. Sgarro A. (2006). A low-complexity distance for DNA strings. *Fundam. Inform.* 73 361–372.

[B10] Drmanac R. Petrović N. Glisin V. Crkvenjakov R. (1986). A calculation of fragment lengths obtainable from human DNA with 78 restriction enzymes: an aid for cloning and mapping. *Nucleic. Acids. Res.* 14 4691– 4692. 10.1093/nar/14.11.4691 3012475 PMC311475

[B11] Esposito G. Tremolaterra M. R. Marsocci E. Tandurella I. C. Fioretti T. Savarese M. (2017). Precise mapping of 17 deletion breakpoints within the central hotspot deletion region (introns 50 and 51) of the DMD gene. *J. Hum. Genet.* 62 1057–1063. 10.1038/jhg.2017.84 28878337

[B12] Flanigan K. M. Dunn D. M. von Niederhausern A. Soltanzadeh P. Gappmaier E. Howard M. T. (2009). Mutational spectrum of DMD mutations in dystrophinopathy patients: application of modern diagnostic techniques to a large cohort. *Hum. Mutat.* 30 1657–1666. 10.1002/humu.21114 19937601 PMC3404892

[B13] Gonçalves A. Oliveira J. Coelho T. Taipa R. Melo-Pires M. Sousa M. (2017). Exonization of an Intronic LINE-1 Element Causing Becker Muscular Dystrophy as a Novel Mutational Mechanism in Dystrophin Gene. *Genes* 2017:8. 10.3390/genes8100253 28972564 PMC5664103

[B14] Greer K. Mizzi K. Rice E. Kuster L. Barrero R. A. Bellgard M. I (2015). Pseudoexon activation increases phenotype severity in a Becker muscular dystrophy patient. *Mol. Genet. Genomic. Med.* 3 320–326. 10.1002/mgg3.144 26247048 PMC4521967

[B15] Han Y. (2020). *Tool for demultiplexing Nanopore barcode sequence data. 2020*. Available online at: https://github.com/hanyue36/nanoplexer(accessesed dateApr 15, 2020).

[B16] Hastings P. J. Ira G. Lupski J. R. (2009). A microhomology-mediated break-induced replication model for the origin of human copy number variation. *PLoS. Genet.* 5:e1000327–e1000327. 10.1371/journal.pgen.1000327 19180184 PMC2621351

[B17] Heng L. (2018). Minimap2: pairwise alignment for nucleotide sequences. *Bioinformatics* 34 3094–3100. 10.1093/bioinformatics/bty191 29750242 PMC6137996

[B18] Huddleston J. Chaisson M. J. P. Steinberg K. M. Warren W. Hoekzema K. Gordon D. (2017). Discovery and genotyping of structural variation from long-read haploid genome sequence data. *Genome. Res.* 27 677–685. 10.1101/gr.214007.116 27895111 PMC5411763

[B19] Kidd J. M. Graves T. Newman T. L. Fulton R. Hayden H. S. Malig M. (2010). A human genome structural variation sequencing resource reveals insights into mutational mechanisms. *Cell* 143 837–847. 10.1016/j.cell.2010.10.027 21111241 PMC3026629

[B20] Krawczak M. Cooper D. N. (1991). Gene deletions causing human genetic disease: mechanisms of mutagenesis and the role of the local DNA sequence environment. *Hum. Genet.* 86 425–441. 10.1007/BF00194629 2016084

[B21] Lim B. C. Lee S. Shin J. Y. Kim J. I. Hwang H. Kim K. J. (2011). Genetic diagnosis of Duchenne and Becker muscular dystrophy using next-generation sequencing technology: comprehensive mutational search in a single platform. *J. Med. Genet.* 48 731–736. 10.1136/jmedgenet-2011-100133 21969337

[B22] Love D. R. England S. B. Speer A. Marsden R. F. Bloomfield J. F. Roche A. L. (1991). Sequences of junction fragments in the deletion-prone region of the dystrophin gene. *Genomics* 10 57–67. 10.1016/0888-7543(91)90484-V 2045110

[B23] Mantere T. Kersten S. Hoischen A. (2019). Long-Read Sequencing Emerging in Medical Genetics. *Front. Genet.* 10:426. 10.3389/fgene.2019.00426 31134132 PMC6514244

[B24] Marey I. Ben Yaou R. Deburgrave N. Vasson A. Nectoux J. Leturcq F. (2016). Non Random Distribution of DMD Deletion Breakpoints and Implication of Double Strand Breaks Repair and Replication Error Repair Mechanisms. *J. Neurolmuscul. Dis.* 3 227–245. 10.3233/JND-150134 27854212

[B25] Miyazaki D. Yoshida K. Fukushima K. Nakamura A. Suzuki K. Sato T. (2009). Characterization of deletion breakpoints in patients with dystrophinopathy carrying a deletion of exons 45–55 of the Duchenne muscular dystrophy (DMD) gene. *J. Hum. Genet.* 54 127–130. 10.1038/jhg.2008.8 19158820

[B26] Nobile C. Toffolatti L. Rizzi F. Simionati B. Nigro V. Cardazzo B. (2002). Analysis of 22 deletion breakpoints in dystrophin intron 49. *Hum. Genet.* 110 418–421. 10.1007/s00439-002-0721-7 12073011

[B27] Rosche W. A. Trinh T. Q. Sinden R. R. (1995). Differential DNA secondary structure-mediated deletion mutation in the leading and lagging strands. *J. Bacteriol.* 177 4385–4391. 10.1128/JB.177.15.4385-4391.1995 7635823 PMC177188

[B28] Sedlazeck F. J. Rescheneder P. Smolka M. Fang H. Nattestad M. Von Haeseler A. (2018). Accurate detection of complex structural variations using single-molecule sequencing. *Nat. Methods.* 15 461–468. 10.1038/s41592-018-0001-7 29713083 PMC5990442

[B29] Shaw C. J. Lupski J. R. (2004). Implications of human genome architecture for rearrangement-based disorders: the genomic basis of disease. *Hum. Mol. Genet* 1 R57–R64. 10.1093/hmg/ddh073 14764619

[B30] Sheng W. Chen J. Zhu L. Liu Z. (2003). Is the human dystrophin gene’s intron structure related to its intron instability? *Chin. Med. J.* 116 1733–1736. 14642147

[B31] Takeshima Y. Yagi M. Okizuka Y. Awano H. Zhang Z. Yamauchi Y. (2010). Mutation spectrum of the dystrophin gene in 442 Duchenne/Becker muscular dystrophy cases from one Japanese referral center. *J. Hum. Genet.* 55 379–388. 10.1038/jhg.2010.49 20485447

[B32] Toffolatti L. Cardazzo B. Nobile C. Danieli G. A. Gualandi F. Muntoni F. (2002). Investigating the mechanism of chromosomal deletion: characterization of 39 deletion breakpoints in introns 47 and 48 of the human dystrophin gene. *Genomics* 80 523–530. 10.1006/geno.2002.6861 12408970

[B33] Tong Y.-R. Geng C. Guan Y.-Z. Zhao Y.-H. Ren H.-T. Yao F.-X. (2020). A Comprehensive Analysis of 2013 Dystrophinopathies in China: A Report From National Rare Disease Center. *Front. Neurol.* 2020:11. 10.3389/fneur.2020.572006 33101180 PMC7554367

[B34] Trinh T. Q. Sinden R. R. (1993). The influence of primary and secondary DNA structure in deletion and duplication between direct repeats in *Escherichia coli. Genetics* 134 409–422. 10.1093/genetics/134.2.409 8325478 PMC1205485

[B35] Vaser R. Sović I. Nagarajan N. Šikić M. (2017). Fast and accurate de novo genome assembly from long uncorrected reads. *Genome. Res.* 27 737–746. 10.1101/gr.214270.116 28100585 PMC5411768

[B36] Wei X. Dai Y. Yu P. Qu N. Lan Z. Hong X. (2014). Targeted next-generation sequencing as a comprehensive test for patients with and female carriers of DMD/BMD: a multi-population diagnostic study. *Eur. J. Hum. Genet.* 22 110–118. 10.1038/ejhg.2013.82 23756440 PMC3865410

[B37] Yang J. Li S. Y. Li Y. Q. Cao J. Q. Feng S. W. Wang Y. Y. (2013). MLPA-based genotype–phenotype analysis in 1053 Chinese patients with DMD/BMD. *BMC. Med. Genet.* 2013:14. 10.1186/1471-2350-14-29 23453023 PMC3599358

[B38] Yang L. Luquette L. J. Gehlenborg N. Xi R. Haseley P. S. Hsieh C. H. (2013). Diverse mechanisms of somatic structural variations in human cancer genomes. *Cell* 153 919–929. 10.1016/j.cell.2013.04.010 23663786 PMC3704973

[B39] Zhang F. Khajavi M. Connolly A. M. Towne C. F. Batish S. D. Lupski J. R. (2009). The DNA replication FoSTeS/MMBIR mechanism can generate genomic, genic and exonic complex rearrangements in humans. *Nat. Genet.* 41 849–853. 10.1038/ng.399 19543269 PMC4461229

