# Corrigendum: Sequence and Structure Characteristics of 22 Deletion Breakpoints in Intron 44 of the *DMD* Gene Based on Long-Read Sequencing

**DOI:** 10.3389/fgene.2021.706540

**Published:** 2021-06-15

**Authors:** Chang Geng, Yuanren Tong, Siwen Zhang, Chao Ling, Xin Wu, Depeng Wang, Yi Dai

**Affiliations:** ^1^Department of Neurology, Peking Union Medical College Hospital, Chinese Academy of Medical Sciences, Beijing, China; ^2^GrandOmics Biosciences, Beijing, China; ^3^Laboratory of Clinical Genetics, Peking Union Medical College Hospital, Chinese Academy of Medical Sciences, Beijing, China

**Keywords:** Duchenne and Becker muscular dystrophy, *DMD* gene, copy number variations, long-read sequencing, NHEJ, MMEJ

In the original article, there was a mistake in Figure 1 as published. Supplementary Figure 1 of the article was carelessly displayed again as Figure 1. The corrected Figure 1 appears below.

**Figure 1:**
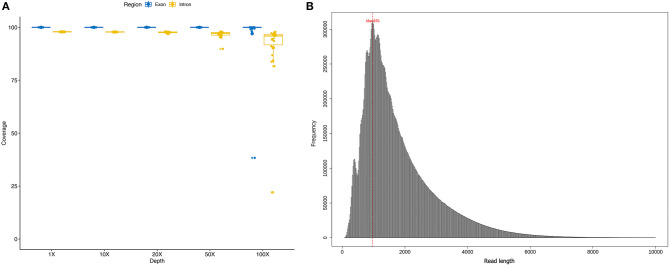
**(A)** coverage ratio in different depth level of each sample; **(B)** the length of sequencing reads of each sample.

The authors apologize for this error and state that this does not change the scientific conclusions of the article in any way. The original article has been updated.

