# Supplementary material for: Sequence and Structure Characteristics of 22 Deletion Breakpoints in Intron 44 of the DMD Gene Based on Long-Read Sequencing
Source: Front Genet. 2021 Apr 30;12:638220. doi: 10.3389/fgene.2021.638220 (PMC8240811; doi:10.3389/fgene.2021.638220)
Supplement: Supplementary file 3 [file Table_3.DOCX]

Supplement Table 1. Special elements in 44 breakpoints of 22 patients

| **Deletion Junction** | **Start breakpoint** | | **End breakpoint** | |
| --- | --- | --- | --- | --- |
|  | **Intron** | **Sequence type** | **Intron** | **Sequence type** |
| N1 | 44 | Unique | 47 | Unique |
| N2 | 44 | SINE AluSx3 | 48 | LINE L1M5 |
| N3 | 44 | Unique | 56 | DNA MER5A |
| N4 | 44 | Unique | 47 | Unique |
| N5 | 44 | Unique | 50 | Unique |
| N6 | 44 | LINE L2a | 45 | Unique |
| N7 | 44 | Unique | 49 | Unique |
| N8 | 44 | Low complexity GA rich | 50 | LTR MLT1H |
| N9 | 44 | Unique | 52 | LTR MLT2D |
| N10 | 44 | LTR LTR37B | 50 | LTR LTR16E1 |
| N11 | 44 | DNA MER1B | 47 | Unique |
| N12 | 44 | Unique | 47 | LINE L1Mb4 |
| N13 | 44 | Unique | 48 | LINE L2a |
| N14 | 44 | Unique | 53 | Unique |
| N15 | 44 | Unique | 48 | DNA Charlie2b |
| N16 | 44 | Unique | 54 | Unique |
| N17 | 44 | LINE L2a | 51 | Unique |
| N18 | 44 | LINE L1PA3 | 52 | LINE L1PA5 |
| N19 | 44 | Unique | 54 | Unique |
| N20 | 44 | Unique | 49 | DNA Tigger4b |
| N21 | 44 | Unique | 50 | Unique |
| N22 | 44 | LINE L1MB1 | 51 | SINE AluSq4 |
