# Supplementary material for: Sequence and Structure Characteristics of 22 Deletion Breakpoints in Intron 44 of the DMD Gene Based on Long-Read Sequencing
Source: Front Genet. 2021 Apr 30;12:638220. doi: 10.3389/fgene.2021.638220 (PMC8240811; doi:10.3389/fgene.2021.638220)
Supplement: Supplementary file 4 [file Table_4.DOCX]

GTCCATGAGATCACAGTACACTGACGTGCAAGGGGCATTGAAGAACATATTTATAGTTCCTACTCCAATGGCTTTGCTACTGTGGGAAAGAATTTGATTT

GGGATTCTTCTTCCCCAGAACCAGTTGTGAGACATCATTTTAGGTGTCTCTTTCTTACTACTCACTCATGCATCCTCTTAGATGGTGACATTAAGAATAG

GTGTTGGTGTTAAAACACAATATATTATGATACTCAAGTAAGAACTCAGTACCTGAAAACAATGACAAAACATGCCATGTGATGTTTATGCTTCAGTTAC

ACCTGTGTGGAACTACTCGCAGAGAAATGCAAAGGATGGAAACACAGTTCATGGGCTTCTGGGTTGATACCTGTCAGTATCACAAATGTGATGGGGCTAC

CCATGAATTTCATATGGATATCACGCCAAAAGGATGCAAAACAATGCGCTGCCTCAAAGTTTTGTGTGTGTGTGTGTATGTGTGTGTGTGTGTGTTTGTT

TCAATCAAACCACCATCACCACCTTCACAACTACCACTACCATCACCAGCACCCAACCATCGTCACCATTATCACTGCCACCATCACCGCCACACCAACT

CAAAGCATATGCAACTATTCATAAAGTGGGCCAATAGTTTGGCAGCCCTCAGTGAAGAACTGAAATCATTTTTGGCTTTCTGTGCCATAAAAACCACAGG

CTTTAATCCGTTGGAGGGGTAAGACTGCACTGTGACATGACTATAGAAAGTAGATTTGTATCCTAGTTCTATTATCCATGTGTGTAAGGCAGAGCATTCG

GGAAGGAGGCACTGGCACAGACACACTGAAGAATCATTAAATTGATTAGACTTGTATGGCATGATGTTGGCAGAGTTTTCATTACGACTCCTGTGTTTAA

GATTCCAACCTAGGGACTACAAAGGATTGCCATTTTAAAGAGATTCTTGCTCCCAACCCCAGTCTTGTCGTCATGGTGATGACAACGGCAATGAACATTT

GGAGCCTTACGAGGCAATGGCTAAAGACCCACTGGCCTTGATTCATAGATTCCTACATCTGTATGCATTTATTTGTATTTCAAAAGTAGACTCTGATGAG

ATGACCAAGAGGAGTCCTTCTGTGTTATAAGTAACTTGCCCTTCACTGGCACACTTTAGTTTGGATGCCAAAGGTGGGTAACTTGTTTCGGCATGTAACT

ATCTTGTTCAGCTGAAAGTTGATCAGCACAGAAGCCTGTGACTCTATACATGTAATGAGTCAGAATGGACAACGAAATAATAGCGTAAAGCAGGGATTGG

TTCAATTTCTACACATATACTTACAAAGGAAGAGATTAGAGATAGACAGATTAGATAGAAATTGGCTAATATTCAGTGTTCAAATATACACTGAGCCACT

TTTGTTTCTGATCTTTGTATGCATATGACACTGTAAATGTGTGTGTGTACATATGGTGGGTGTATATATACATGTGTGTACATAAACACATGTACAATTT

TTACAGCTAATTAGGGTGGACCAGAAACTCAGGTACAAAAAGGAGTCTAGGAAGTCAAGGCTGCCTATGAAGAAGCAGAGTCATGAGCCTTGCTGTAGCT

CCCCCAGAAACGAACAGTAGCAGCAAAACATCATCTTGGATGAAGCAAGACATCTGAAGCTCATGCAAGCCAGTGGGAAAATTCTGCAGGGTAAAATGCT

CCCTTGGAGAAAAAAAAACTGTATATCTTTTTTTTTTTTTTTTTTTGAGATGGAGTCTTGCTCTGTCACCCAGGCTGGAGTGCAGTGGCACGATCTTGGC

GTGCTGTCTCCTCAACACAAAAGAGTAAGAATTGAACACTTTGCAGTCACTTCATGAAGGTAAGAAGGGAGTTGAGACACTGAGGTCTTTTTTTCACTCT

GAATGAAATACTACCAAGAGGATCAAGATCCTGACAACTGCAGGGCTGATTACATCTCTCTGTTTCATTACTGATGCAAATTTTTGCGGAACTGGGAAAC

GCCACCACGCCCAGGCTCATCTCTTTTTAAGAATCGAAATCAGACATCACATATGTGCATGAGTAGAAGTGAATCAACTCATTGAAAAATGCTTAATGCC

CCAGCTGATTGCCAGCAAAAGTTCAGCCAAACAACAGATGCGTGAATGAGAAATATGCAACTTGAGAATTTTGTGGTTATTTGTTATGCAGCAACAGCTG

CAACCAACGAGTGGATAAAGAAAATGCAGTATGTACGTGGTGTTTATATACACACACACACACACACACACACACACACACACGCCATGGAATATTAGTC

CTGACTCACTTCCCTTTGATTCAAACGCTGATAATGGTTCTATCTGCTTAAAAAGTGATTTTAGCCAGAACCTACGCAGCCTATGCAGAATAGACACAAG

TGGTAGAAGAGGAACTGAAGTTTCTGGAAGATAGCTTAGAAAGGGAGAGAGACCAAAGGCAGAGGAACCAATTAGGAAAGTATTCATATTGTCTGTTCAG

GGTGAAGTGTTATGTATGAAACCTAACCATTGAGTGGATCAGTGAAAAAAAAATCCACAAACTATATACACGCATGAATATGATTAAATATGCACATAAA

TTATAGCAGTGTGAAAACAGATTAATACACAGTTCAAGAAAAAGTACCCTTGAATTCAGTCTTTTCTTTCAGAGGACAAATTTAACACAGCTTATCCATT

CCCTCAGTGATCTCCAAGTAAAATCTTCAGCAGCTCGCTGGGAGGCACCATGTTAGATGTAGAAATTGCTTGAAAGTATTTTACTTCCATGATATCCAAC

CCATTACCCAAATGGAGGTCTTTTCCAAGGCACTGGATCGAAGCACCTGTGTTATAGATTTTATAGATTTCTCATCTCACACCCTTCAGCAATCAATTCT

GAAGGAGAGGACACCGTTGTGCCATTCACTTTGGCCTCTGCCTGGGGCTAAGTCATCCAAAAGAAAACAGAATTACTTTTCATAATAAACATACAAATGT

TTGTGTTGACGCAGTAGCTTGGCCTCAGCAATGAGCTCAGCATCCCGGGGACTCTGGGGAGAGGTGGGCATCATTTCAGGAGGGGACGGCAGTGGGGACA

ACAGAATATAGTTTGATGTGGAATTGTTGGATCTTAGCAACATCTTCAACATTGCTACATGTCACCGAATCGCTCTGGGTGACAAATCGGTGCTTTTACC

CACTCCAGCTTTAAATCAATTGATGTCTGATAGGCCTTCTCAGCCCACTTTGTTTTTCCTCAAAATTGTCTTGACTATTCTGACCCCTTTGATCTTCTCC

AATAAATATAAACAGTATTAGCACATGTGAAGGTCCCAAATGCTTTAGGATTTACTGAGAATACAATTCCAATTGAGGACATTTCTGATATTTGATATCC

ATGGTGTTCCTTTTGAAAACAGTTTTTAGACATGGACCTCAAGATGGTACAACAAGCCATTACTGTCTTCCCACCTATTTTTCACAAAGGTGCTGACAGG

TCTCAGCCTCCCAGAGTGCTGGGATTACAGGCGTGAGCCACTGCACCTGGTCTGAGCATCTTAAATGTAAGTAAAAATCTTTGTTGTACTAGTGTATTAT

AAATAGTAATCCTGGCTTTGGGCCACTACCTTGTAAGGAGAATTTTAATCCCTGTTACTACAGCACGAGCTGAAAGCCCACTAATTTAGCCCCACTTACT

CTGGAGTGCAGTGGTGCGATCTCAGCTCACTGCAAGCTCTGCCTCCTGGGTTCACATTCTCCTGCCTCAGCCTCCAGAGTCACTGGGACTACAGGTGCCC

CAATGTTTTCTTAACAGTATCATACATTTAACTGTATACCAAAGCATGAGCACACACAGGGATTATTTTGTAAAGTAGAACAACTACAAAGATTGCCATC

AAGAACGTAAAGGAAAAAAATAAAAGCTTTGCCCCATCAGGGGAAAGGTTGAACAATGCCACTTTTTGACACGCTGCCTTTGTGGTGCCTTTCACCATCC

CTGGAAATAAGACAGACTAACAAAACCTAAGACAAAAATGGCAGTGGCTGGTCATCATCAGCCAAAAGGAAAAACTAGGATTGGAGAGGTTTTAGAGGTG

CAAATCCATAGAGACAGAAAGTAGATCAGGGATTGTCTACTGCTAGGGCTGAAAAGAGTAGTTGCAGGAGAAACGAGGAGTGACTTGAAAATGGGGATGG

GTAAAAAAATAAAATGGGTGAGTTTAGCCATCTGCAGAAGCCATCCATGTACATATGTAATACCAACAGGAAACCATTTCTAACTGGGCTGCTCATGCCC

AAGTTACTTTTGTGCGAAAAATCCAGGCTTCCTACGTGGGCACCTGTTATAGCTTTATCTTCTGATGTTCCCAGAGACACAACGGATAGGCTTCTCAACA

TTCCACTTGAAATGTCCTTGGGCAAATAGAATTCAGTATGTCTGATACGTAGCTCTTTCTCTCCATGCACTAACCCTTTGCAAATCAGGAGTTTTTCCTG

AAGGGAAATTGGATTGTAAGAACATAAACTCTTATCCAGCATGTTCTAGGGAGATGGCAGCTGTACTTGTAAGATGTGAATATTCTAATCGCCAGAGGGG

CACCAAGTAACTTGAATGCCTAGTTTCGTTAAATGTATCATATACCACGCTTTCCTCTCCACTCCAACTTTAAAAACATGTGGGAACAGCCGTAGGAATC

ACCTTCCAGCTACTCAGCCCTTCAGGAAGGACCTCTAATAAACACTGCTGCATTAAAGGATGGACCTGCTGCAACTCCTCCTGAATTATCAGTCCCAGCT

AGCCACCTTGTTGACTTGAGAACAATCTAATCAATTTGCTCTGCAGGTAGTAGAACGACAATGTCTTTGTCTCTCAGAAAGGCTACCTTTTCATTGCTAG

GGGGGTTGTAGGGAGGTGTCATGAATAAGAACAATTAATGGAATTTAATAAATATCTAGTCAGGAATGGGGCTCTCCAGGCCTCTCTCTTTTGCTGTTAT

ATAAATGATAGTTATCAGTACTCTTTACAAACATTCTTTGACCCGGGTGCAATGGTACGGAGAAAGACTATGCTAACCACAAACGAGGCAGGGCTCTTGA

AAGTAGAGTCAGTATGGAGGATTTATTGCAAGGGTGGGAAAGAGACTAAAGGGAAGCCAACCAGATAAGGAGCTATGGCCATAGGTAAAAGGTGTTAAGA

ATTCCTGGCACTTTTCTATGTGTGCAAGTGTATGCACTCTGCATACCAATGACAAAGCTAGAAAGAAAACTCACCTGTTTTCTTCCTCAAGATCTGCTAG

GCAGCTACAATCTTTCCTGGAAACTAGGAACTCTCTAATACACAGGACAAACTTTTTTCTGAATGCATTGCTCCAAGCTCCGGAAAGGCCTGTTTACCTC

TATTCACTTGGCAAATCCTTATTTTTTCCTTCATATTTGCTTCAATGTCTGCTCTCTCTACCTCAAGCCCCGGTCAGCATTTACTTGGTTCATGGCAATG

ACTAAAGGATAAATCATGGCAAGAAGAGAAAGGAATCCTTCAAATTAAAGGATGTGTGTGTTGGCTTAACCATTTGAGGCAGTGTGTGGTAGACCACAGC

GGTGACCAAAAACAACATGCAGTGGAACGGCATGCACGTTAGAGGGCAAGTTGATTAGTAGGAATCAGACAAGTTTGGGGAAAAAAAGAAAAGATTTGTT

ATAGCACAAAAGGTCTCTTCCATTTCTCTGCTGACACAGAAAGCCCTTAGGTAAAGGCATAAATCCAAAAGTCAACGATAATGTAATTCAGAGAGGACCC

AAAATCTTATACAGAGCTTTAATTCTTTGCCTCAAGGATAATTCCATGTTATTTTTCTTATTAATATCAGTGGCGCCTATCACAGCATGCATAAGCACAA

GACAGACAAAATCTTCTACTTAAAATAAGTTAATCTGATTGTCATAATGTTTTTCTCATTAGCTTACGTGTAGCTCCTTTTTGCCTTTCAAGGACTATTA

CCCTTGTATATAATGATTAGTCAAATAGCATGGCATAACCTGATAAACAGTCCGCACACCTCATTAAAATTATTCAACCAAACTCAGCATCATACTTTAA

GAAAGAAATAGAAGAGACTGTTTGCATTTGGGAGTGAAGGAGGGTGTTCAGCTGAGAGGAGTTCAAATATACATCAAACAAGAGTGTGTTCTGCTTTTGC

CCAGCTGCAGATCTCGCTAAAATGTGGGTTTTGATTTAGAGCATCAGGAGTTGGGGCCTGTGGTTTCACATTTCTAATAAAGACCAAGGTAATGCCAATA

CCGGAGTGCAATATTCCACCATGGGATAGTGCATTTTATGGCCTTTTGCAACTCGACCAGAAAAAAAGCAGCTTTGGCAGATGTCATAATTAAAGTGCTT

GGGACAGGAGGAATGAGAAGTGCTGGGTCCTAAAATAATATAATATGATAAGGGCAAAAGAATTGAAGGTGAAAGTGCTCTTTCAGATCCTCCAAGGCAT

TATAAACAGGAAAGACCGTGAAAAAGGAAAGATGGTAAAAATGTTTTGGTTCCAGGAAGGAGGAGGTACAGTTCATCTGTAATACCTCCGTGAAGACACG

TCCAAAGCCTCATCTAACAGACAAGCAAACTGATTTTGTAGACCAGAAAAGGAGCTTTTCAAAGGCCATACAGCTAGCAGCAGAGCTGATAATAGAATCC

ATATTACTCCACTTTCGTCTATCTCTGTTGCTGCTGAGAAGTCAATAGTAAAGATGACTGTTGCTCCTTTGCAAAAAACACATGAAAAAATGCTCACCAT

CCCTAGTTCCCCCCTCTCTGCCCCACATCTTTTTAAAACATCCTTCTGTGCTTCATTCTTATTAATTCCTTCTGCCTTACTGTCTGGGTATCCATTTCTC

TAGCTGCAAAATGGTATCAGTTCAAACTTCCGATCAAATGCTACTGTTTACCAAAGACCTAGAATCAATCTAAATGCCCACCAATGGAAGACTGAATAAA

ATTCTTCAAATATGATTGCGGAGAATGTACCTCTATTTCTCCAGACAGTGTAACTGGATTACTTTCTTTTGTTCTGTACCGTAGGCTCACACACGGTTCC

GTTGAGATACATATAAGTCTCAGATATAATCCCCTCTCAGTATCAAGGGTGACCTTGTCCTCATCTCTAAACACCAGTTTCAAGCCATGCAAGCAAAATC

GCAAAATCCACGCATACGCAAGTCCCACAGTAGGCCCAGCTGAACGCATATCCGCAGGTTTCACATCTCTTGAGTACTGCATTTTCGATACGCATTTGGT

GGCAGATGTTCTTTTGAGAGCATATTACGTGCACTGCTGCAGTCCTAACAGAGGAATTTCTTGTGGGGCGATTTTAGAAAGTCCTTGAGACAGTCTTTAT

ACAGAAAAAGGTAAATTTATGGAAAACATTGTGGGCAAACCACAGATTTTTAAAAAGGGTAATCTCTCCTTTGTGGCTGATATGGTTTGGCTGTGTCCCC

TGTACGGACGCAAGGGATCATTTACATGAGGTTGTACACAATGGTTTTTCACATCTTGCTCTTAGTGTGTGTTTCCATTCAGAGAACACTTGGTCTCATG

AGAGAGATATCATTCTTTGTACACTCAGAACCACCTTGGATGTCCACAGTGATATTCATGAATAGACGGGGGAGAGGTAGAAGCTCTTCTTTACATAAGA

GGGAAAGTTCAGTGTGGACTGCATATCAGAGGGTATTGCTGGATTGCTGTTAAATTTGTGTGTAACGATAGCATTGCAGTTATGTTAGAGAATGTCATCC

GCTCTGCACTCCACCTGACTAATAAACCAATTAACGCCATGGCCCTAGGGAACATATGCACAATTTTAAATCTATGCAAATCCCTAAGTATACTCAACAG

CTTAGTTACTAGTGAGTATTCATCCGTCCAAAAGGGGTGTAAAAGGAATGTCTATGGAACCAAAGGGCCCACAGTCTGATTAATACACAAAAAGCCAAAG

CTATGCACTTAACCACTCTGCCAGTCACTTCTCCAGATATTCTGGAATGCTTTCTCATTTATGTGTTACTGATCCTCCCACCTCAGTCTCCTGAGTAGCT

TGGGCGCAAGTGATCCTCCCACCTCAGCCTCCTGAAACGCTGGTCCTACAGGTGCGTGCTACCATGCATGGCAACAATAATTTATTGTATATTTCAAAAT

TAGACCTGATAGGTGATTTCCTTTTCTAAGTGGTTAGTGGGGAGGGATATGAGAAAACCTTTAATAGATAATCCAGAGAGTGTGGCACACAAACACGTAC

CGGTGGGCATACTGGACTAGGAATCAAACCCTTCCACCAGCTGAGGGACTTTAAGGGCGCAAATAAATTATTCATCCACTTAAACAATAATAAAAGCAAA

TCTGAAAACAAAACAAAACACATTTCACATTTCAAAGAGCTTGGAGACTGGCTGGCCAACCAGGAGGCATTCAACAAATATGCAAGGATGGACTGAATTC

CTATCTGCATTTTGGAAGGTTTGGGTATGAGGAGGAAAAGATATTTATTTCTCCACATGCATTTTACATTTACAACTAATTTGCCATAATTTTGGCAAAA

AGAAGAGGGTGTTTCTGGGGCACTGACTACGACAGAGAAAAAGCAATGGGACAGGATAGGAAAGAGAACGGACAAGAGTGTGTTCAGTCAGTGATGGACA

GTGTGATAACTTTTACATAAACTGTGTCACTGCAAGAAGCGCAAGCTTACACGTACCCTCGACACTTAAATTTCTTCTAAGCCAGAACATAATCCTTGGG

GTTATGAGTTACTTTCAAACATCTCATTTTGCATTTAATATTTTTGTTTCACCAACCTAAAAACATGTTCCCTCTTAGTAGTTATTCGAAACACCAACTC

ACAGCATTAATATACACGACTTACATCTGTACTTGTCTTCCAAATGTGCTTTACACAGGGAAATGATGCCAGTTTTAAAAGACAGGACACGGATCCTCCC

GCTAATTTTATCCATGGGGGAAAAAAAGGAAAAATAAGAATGAAAAGTTTTCCATTTTGTAGAAAATGTCTTTCAAGACATGGAATCAATATGAATGCCC

CACCAAATACTTTTCAGTCAGGTAGAAGGAAGTTATACATTCAGAATGCATTCACATGTCATCTGCAAAGAAAACAGTGCCAAAATATATTTGAGGGGAA

GATTTATCACAGGAAGAGCATTAGGTCCACAGCTTCCAGGGCCCTGTTGTAATGCTAATTACACTTCACCATTCTGTACGCTAAGCCTCCTGTGACAGAG

GGCTGAGGGAGGAAAATTCCCTTGATCTGAGGAGGTGTTTAATCTTATGCTGGGGACAAAAATTTAACAAAAGTGAAACTTCAACCTTGTCCAACATCAG

CCAGCTCCATTACTGGAAGAGAGGAATGGATTTCTTCCAAACACCAATATTCCCAACATGTTTCCTTATATACTGAAGGAAGCTATGGGTGGTTTGAAGT

GTGAGAGAGGGTGGGGACAGGATAAAAGAAATGGCTGACTCTCCAGGATCCTCTTTGCTGTGCAGGAAAAGCAGACAGTGGCGGTTGGGGGGCGGCGAGC

AGTCAGACCAACGGAAGCCACTGAGTTTGAGGCTGGTTACATAGGAACAGTATGCCTAGGTTACCCACGGGATATAAGAAACCCTGGCTGAAACTCCAAT

GCATTCCACAAGGATCAATGGAATGGATTACTCCTTCCTTGTGCCTTGCCTAATTGAAGCTATCACACAACCCCATCATAGGTTGAAAATATCTTAAGTC

AAAACCTCCTCCTCCTTGAAAGAGCCAAGATGTGAGTGGGAGAGTCAGCCAACCTATCCAACACACTGGATGACGAGATGGGCGTTCTAACTGCGACTAC

TATTTCCCCTGGGAGATAGATGGGTCATGTTATGAGACCTCTCTTGGGCACTAATATCTGTCAAACCATATAAACTGAGAAAATTAGTTTGGCCATGTCC

AGGGGCAGGGGCAGGGGCAGGTGAAGAAAAAGAGGCAAGAAAACTTTCTCTATTCAGCCCCCTGACTCGATCCCATCCCAAACTACTTTTTCCCAGCTAT

AGCCGCCTCTGGGTTCACACAGCTGGTTTTCAACTCCGCCCTCTCTCTTATTCTCCACAAAGATAAGCCCAACTTCATCATCTCCTGCTGTTAACTGCGT

TAAAATCTCACCAAAGTTACTAGGGACCTTCCTGTTGCTAAGTCCAATGGGCATTTTCCGCAACTTTATGCTGACATCCCAGCAACATCTGACCCTGGTA

TTAGCCATACTGACATATTTTCAGTTCCTGAATGTGTCATGCTTTCTCTGGGCCCTTCAGCACATGCTCTTCTTTCTGACTAGAACGCTCTTCCTAGAGA

GCAGTGAGCCCATATTGCGCCACTCCACTGCACTCCAGCCTGGGCAACAGAGCAAGACTCCATCTCAAAAAAAAAAAAAAAAAAAAAAAAACAGAAAAAA

GGTATAGCCTGAAGGGCATTCATGTAGTCCTGTTCCCAAGAGGAAAGAGATGGACAGGCATTATTTTTTTCTAGAAAGTAATCTGGATCTACTGTAGTGA

CCATATCCAAGTCACTTGGGTCAACATTTTCAGCTTTATGGCCATCGCAGAGACCAAACATAAAAGCCTGAATTTCAACTAGGGGAAAGAAAACAATGTC

GCCACAGGCGGCCACTGTAAGTGATTCAGTTATATTTAGCTCTTATTACACCAGGGGTTTCATTAATTTTCCTAATTAATAGTTAGACGGGCCTGTGTAG

CAGATTGCTTCTTGAAGATAATTAACATTTGCCTGGACGCTGTCCTTTAGTGAGCAGAACTTGATCTAGTTGAGTCCCTTTAATTGTCCTTCTCTCTCTC

TTCTAACTATGACACATTCTGCCTCTAGTTAATCCTCCCTGACATCTTTTCAGCCTACCCCCAACCCACTGCAGTGAATAAGCCTTCTGCTACCAGTTTT

TGAACTGCAACGGAGTTCTGGTGCCCTACAAGTCTACTACTCAAGAGAGTTAACTGCCTGTGTTGTTTGAGTCCGCATGTGTTGCTAGGAGTCTGCAGCA

AAACAAGGAGTGTTTTAGTCTATCTTTCCAATGGCTTATGGAGACTTTTTGGAAAGGCCTAAGGTTTCAATATGCCTATCTATTAGCTAGAAAGAACAAT

CAGGTGTGGTGGCTCACACCTGTAATCCCAGCACTTATGGAGGTCGAGGCGGGCGGATCACGAGGTCAGGAGTTCGAGAGCAGCCTGGCCAACATGGTGA

GTGCATGTACTATAAGCCAAGAACCATGCCAAGTACATGGAATACACTGGTAACCAAAATAAATCTCTGCCTTTATTGACTTTGCAGTGTAGCGAAGGAG

GGCATTTACCCAAAAGAAAATTAACAAAACAGGAGAGGAGATGAGAACAGATCAGAGCTCCAGTATTTTGGTGGCTTAGAAAATGACCTGCTGGTAACCA

CCTTCAACTCTATTCTCAACCTCATTAATTAACGAATCTTAACAGTCTCTCTTCATAAGTGAACACACATCCCAGATTCGCCAGGGCAGAGCCAGTTTAA

GGAGTCCCTCCAATAAGTAGCTATTACCTGGCTGTCACTCAAATTACTCTATGGAAACAGTTGTTACTGGGTTTTCCCAAAACTTTCCAGCGATTAATTC

GGCATTGGAAATTTCCTACATAAGCCATGTTACTCATGAAACAGTAATTTCTAAGCCCACAAAGGCTTAAATTTAGTTTAAATATCCAGTATACAATAAA

TAAGCAACAAAATGCAGAAGACTAAATAGGGAGCATCGTCCACCCCCGCAATCCTCCTTGCGGTGGAGAAATGACGTGAGCACTAAAGAGATCTTGGGTG

GGGGAACAGGTGGTATGACTCAAATTGCCCATGCATGCAGAAGCATGTGATAGAGAATGTTTTCTTTTCACTGTTATGTGTTAACTTTTCTGGAGACTAG

ACCTAAAGCAGCCCTTTTTTCCTAAGATGTAGGTGACTCTTACTTCAGCTCTTTTTGTATGCAAGTATTCCCTGTAGAGCCAAAGAGAAAAACGGAAGTG

GGACTAGAAAGCTCTTAGTAACTTGTTCTGAATTCCCTAGTCAGCTAGGAGAAAAACATCAACTAGAATCCAAGTCTTTTTCTCCCCAAGTGTGTATCCC

CAAGATTTCTCTATCTGGGATTTCTTCCAAATTCTTAGCTTCACCCGCTGTCCTTCAGGAGAGTACTATTGTACAATGGAAAAACTACTGGGTAGAGAAG

GGCTAAATCGCTGGTTTAGGAGTTAAAAGTACACGTAAATCCCTGCTTTGCAATGTGAGAAAATTACCTATTTTCTCTGAGCCTCATCTGCACAATGGAG

CTCAGAGAACAATGTCTTCACTGACTCGCAAACAAGCCAGTCTTTCATACCTTTGTTTATGCTGCTACACTATCTGGAATATCTTGGCCCTACATTTTAC

GTCTTCCTCACCCAAGAGGCAGGAAGCAATCTTCCTTTTCTGTTAATAACTGAATAGCCTTTTATTAGGACAGCCACTGTAATTTAGGGCTCTGCATATA

ACTGTCTGTTTTTGACCACACTGACTTTATTCTGTAACATCACTTATACATAAGTAGCTTGTTGGACAGAAGGTATTATAACACTTTACTTGGCTTTCTT

CCTTGCTCTACTGTGCTTCATTCAATGAACAATTATTTTTTCAGAGGCTACCATGCACCAGGCACTGGTGACAAAGATGAGGAAGACAGTTCCTGCTTTA

GCAGCAAGCATTGATGCAGAAGCTGCAGCCAAGTTTTCCAGATGAAGCTAAGGTCATGGATGAAGGTGGCTACAACGAACAACAGATTTTCAACATAGAT

CCAGGAAGGCAGAGGTTGCAGTGAGCCTAGATCACGCCACTGCACTCCAGCCTGGACAACATAGCAAGACTGTGTCTCAAAAAAAAAAAAAAAAAAAATC

GTTTCTTGTTCCCGAAATATCTGCATAGAGGGTTGGTTGCCTTACTGTCCATGGGATCTCCTGTACTTCAATCATTTGCTTGTTGGTTTTTGATACTTTC

TCCTGCCTCAGCTTCCCGAGTATCTGGTACTACAGGCAGACGCCACCACTTCTGGCTTTTTTAAATTTTTATTTTAGTAGAGATGGGGTTTCACCGTGTT

AGCTACCAAGTGGGAAAGAAAATAGATCCCTCCTCTGCCTGAACTTGACTCAACTGGCAAAACATTAAACCGCATAAATAACATTTTCAGGTGTTCCTGT

CTGGGACTACAGGTGCGTGCCACCATGCCTGGCCTTAAAGTGATAGCCTCTTTACAGACCAAGAAAGTAAAATATAAAAACAATCACTTTCTTACCAAAA

GGACTTGGTTCCTTATCTTCTACCATAGTGAGTATAAATCAAATCAAGTCTTGAGTCTCAGTCTGCTCAAGTCCAAATGGAAAGAAGGGAAAAGTTGCCC

AATATGCTGAGAAAAGCATTAGGACAAGCTCCTAATGCAAAAGGAAGGAATTCATAGTCATTCAGAATCCTGCAGTTTCTAACCCAAGAGCATCCTCAGG

TTGTATTTATAATTAAGAGACCCCCCCCCAATAAGACAGGCTAAGAAATAATGTTTTTAAACAGGGTCTCGTTCTGTTGCCCAGGCTGGAGTGCAGTGAT

TACTTCTAGGGAGTGACGAAGAATGTACCCTCTCAATAACAAATGAATGACCCAGAGGAGTCTAATGGGAAATATCTCAAGAGCCTCTCTATTTGACAAC

TTATTTCTTTACTATGTCTTGCTTTGTTTGTGTAGGACTCTGGCAATATTTTCTCTCAATTTTTTGTGTAGGTACACAGCCGATAAATGAGGGAAACACA

TTAAACATTTGACACGCCAGACCACAACACCATTTCTGCTTTCAAGATACCTTTCACCAACTGACTGTAGTTGAAGGCTTCCGACCATTTCATCCAGCTC

GCACATCCCATGTGGAAAACATACAATAGGCCCGCAATAAATGGGAGATGTTATCTGATGAATCACCACTTGGATAAAAGCCCAACGAATGTTTGATTGT

CCCAGGACAGCTGCTGCCCACTTCAGATGAAGCCTTTTCATTCTCTCATTTGCCAAAAGATAAGCATTCACGTTTTCCACCTGTGGTATAAACAGTCCAC

CTGTCAGGCTCTTACATAAACACAAAACAAGCCATACTAAAGAAATCATTTAAATGTTCACATTAATGACCCGCCAGGCCTTAGGAATTGTGAGCACCGG

CAGGACTTCTTCACCCATCCAATGTGGAACACTACAGAAAGGACACCGACAACTGATTATTATTCAACTCTGTCAAAGAGATACCGGACATTAGTTCAAT

GGTAAGTGACCGGCACCATTTGGTGCTCAATATGTGGTCGCTTTTGGTATCATGTGCCCCTCAAATTGTCATCTATTAAAAGCAAATCTGTTATTAGTTG

GGAGAATCCCACATAGAATGAATGAGGGCTTCATGGAGCTCCCCATCTTCTAAAGAAATTTCATGACTGTTGGCATACATACATTCCTGCCTTTTTAAAG

CCACATCGGAAATACACTCCCCATATACCATGAACAACCAAAAGGGAAATTCTGAAACCTGTTCACATGTTAATGGGTACATCTTTGTTTGGCCTTTCTG

TGGAGGGGTGGGCATCAATATAAGCATCAGAAATCCATTCTAGTTCCCCATTTCTTGTTCCCTGGGATATTTACCATTTTATTCTGTCTGGCTAAAGCCA

GCAGTGCAATGGAAGGTAGCGCCGCGTGCGCCCCTTCCTTCTACGAGTAAGAGCGACGATTTAGGAACAGCCAGCCTTGCTCACGTCCACACTGGCAGCC

GGCCGGCGATAAGGACGTGAAATTGCCGTTAGGCTCATCCTGGGCTAAAAGGGATTGTTAAAAAGACTTTGGATTTGGATTTGTGACATAGCAGCGGATC

TTTAAGCAAATATGGCACTTCTTCTGGCATTCTGGCTCATACACTGAGATTACTTAGTGTTGCCCTTTAGCAATTTTAAGCTAGTGAGTTGTGGAGGCAG

CTGTTTTGATATGAACCACGGACATGAAAAGAGCCTGAAGCTCTGCTACCAGAACAAACTTCAGCTAAAATTTTGACTTCAGAATCAGCCATGACCTACT

TAGAGGAGAAAGTCAGATCCCCCCACTCTGGGGCTAAAAAGCAGATGAGACTCAGTCATTGGATAAATATTCAGAGAAGCCTCCTCTGTAGGCCCACAAA

AATGAGCCCATCTAGTGGAGGCTGTGAAGGGCCAACAGCTGAAACTAGGCTGTTCCTACCTTCCACAAACACATCTTTTTCCTTTCTGGGTTGCATGTGG

AATGGATCACTGGTTAATGTGGTTCAAGTATATCTGACTACAGCATTCCTGGTCTACCTTAACAGCTCAAACTTGTAACTATTTGTCTGCCTTTCCCCAC

GCTTTTAGAAGTAGTCATCTGTTACTGTCAGAGGTTCATTTCATTTTGAGTCTTTTTAATTAAAATGCAAAGTTCTACATACATGCCTGCCTAAATTACC

GACAGCAGAAACCTGGAGTAGTCAGAGAGCAAGTGAATATTGATTTGGTGGGGGACAGTGAGGGAGAAATATCCCCTAGCTTGATGGAGAAAGTCTCCTG

GGGAAGGCTAACTGGCAGAAAATACTACTTTCCCCATTAGGACATCAAAGCACATTATTGAGTTAATATGAACAAGACCTTCACGTGGGAATTGAAGTCC

TTTGAAGATCACAGTGCATGATGTGGTTATGAAAATTTTGCTATGCATACCCACCAGAATGAGCAAGACAAACAACACAGGGTTAGAAAGGATGTGGAGC

AAGGAGGAAAGAGTGTAAGTGAAACAAAGGAAAATCCAAGTCTGGAATGACTTAGATTGAATAAACAGATCTGCATGGGATCTAAGGTGGGAGACACTGC

GAGGAGGATTAAAAGATTCAGGAGGGGGAGCAAGTTGGGGAGGCGTTCATCAAGTCTGAAGTTCTAACCACATGAGATGCTGCAGGGGCTGAGAATCTGC

ATCTTGCAACATGGCCAAAAGTTCTAGCCCTCTCCTTGGTGTCCAGCTCTTGCAGTGTGCCTTTGTATCTCTTCCCACCAACAGGTGGAGTCTTTCCCCA

TAGAAAATTCTTGAAAATGCAAAGTAATACACAGTGAGAGAAAGACGATCAGTGGTTGCCTAGAATCAGGGGTGAAAGGAAAAATGACCTGCAAAGGGGC

CTAACCATCAAGCAGCCATAAAAAAAATGAAAATGAGGTGCATGTGACATGGCAGATTTTGAGAGTATCACGCTGATTCCCAGCAGCATGTTATCCTATG

ATCACTTCTCTGTGCCTCTACCTTCCTACCCTTTGATGACACCGGTTATATTTCAAGATTCTCAATGCTATAAATGAACACTTTACTGAAGAAGCATCTT

GCCCTCCCCTACCCCAGAATCCTGCATAAGAATGTGAATGGTTATGTTTGTTTGCGCAGATGGGGTTTATATTTATGTGTTTCTTTTCCATCTAGCAAAT

GTGATAGGATCTTCAGACAGAGAGGCTCTAGGGACTTGAAACCTGGAAGTTCAGCATAGGCATTTAGTATATGTTGAGCCTTAGGGAACTCATTTATCAA

CATATTTAGGCTGTTAGGACCATTCCACTGTCCATGGAGGTGCCATTCTGAGATAACAGGGAAGTGGGCAAAACTAATACAAATTAAACCATTGTCAGTA

ATAGTCTACTTCCTTCCTTCTCAAAGTGTGGTTGGTCCACAGATTAGCAGCATTAGCATCACCTGTTAGAAATGCAGAACATCATGTCTCATTTTAGACC

GCAAGAGAGGAAAAGAAAAACACATTCTAAGTGGCAAGTAACTGGCAGCAGGCACTTTGTATAAATACCAAAAGTAGCACGCGTTAGCACATTGATGACG

AGGCCAATTGGTATCTTCAATCTTTTTCAAAGGCTTAAATTTAGGCACATCCAGGAGTAAGAAAGTCCTTCCCTTCACTACGTGCAAGATAAAGCTGGTC

AAACAAGATGAACAGCAAAATATAAGACCAGGCCTGAATCCACTGAGCTGTCTCCTCTATGAACTTCATAAGGCATCTTACATAGAGTAAGCAGACAGAC

GTGATGGTAGTATTAATAGTAATGAGCTAATACCTCATCCTGAAAATAGCTCAAAGATTACCTTAAGTACATATTGACTCCCTAAATATGTGAATAACAA

ACAATAACTTTCCGGAGCCACCATTCTATAACGGCACATGTATTCTATAGTCCCTACCAGCTCCCATTAACTTACGCTTCGCTAGCCTTGCCCAGTTACA

GGAAAACCAGGAGTATCTACTAAAGCTCAGCATATGCCTACCCTCAACATAGGCAATTTTACTGCAAAGTATATACCAAACACAGTTGTACACACGTGTG

CTACACCATATTTTCTGATCTATTTATTGAACAAGGATACCTCAATGTGGCAGTGAGGCTTGGGTTAAAATTGTTATGTCACAGCCTCACCTGCTGGTTG

Supplementary Table 4. Probe sequences of the first 200kb of DMD gene

AACACATGGGGCTATCTATAAGTTATCTACCTGAATATATCCTCTACCCGAACATCCATGGCATTAGAAAGACCATGGGATCAGGTATCAGAAGATCTGG

ACCATACGATTCGCCCATTTAAGGTGTACAATTCAATATTTTTGTTTGTTTGTTTGTTGAGACTCGCTCTGTCACCCAGGCTAGAGTGCAATGGCATGAT

GGCAATTTTGTCACAAAGATTTTTATGGGAATCTGCAGTATGTAGAAAGATTCTCCTGAGGAAACGTGCTTACTGCTCCCTTTACAGTAGGTCAAGAGTC

CGCTATTCCCAGAGTGATAGAGCAAGTGACTAGTAGTGAATGTGAGGGACCTCCTGTTTGTGTGGTTTGTCTGTCATATAAATCTTGAAACTTACATAGA

ATTAGAATTCAAACACACACACACACACACATACACACACACACACACACACATACACACACACACATACAGAGTAATAGTGGTAATACCACTGCCAGAG

AAGTTGGAACCTAGGGTCAGATATTTCTGAGTAAGAACTGAAGAAAAGTCAACTAATTGTTCGTTTAGAAAATAGGGTAGGCTGGGTGTGATGGCTCACG

AGGGTCTGTAGAACTATCAAATTACCTGTGTTATTGAGGTGAAAGGTGAATTTGTGGTGGCCTTATTTGTCTACCTGATATTAAGCCAAGGGTACTGCAC

ATTGGGGAAGGGTCACAGTAGCTAGTCACCATGAATCTGCTTATACCCTTCTCTTCAGAGGAAAAGACATCACATTGCTATAAAGAACTACCCGTGACTG

GCTATCTAAGATGCCTGACTTTTACCCCTGAGATACAAAGCATGCCTAAGCAAGAAAAATCACAGTCTAAGGAGACAAAGCAAGAATCAGAACCAGACTT

TGCCACCCTAAATCTGATCTTTCTTTAGAGTTCCCTAACTCACTGTAAAGAATTACCAACTGTGCATTGCCCAGAAATCTAGGTGTGATTCCTTGACATG

CCTATATTACAGGCCAATTCAAGAAAGTAAATCACACAGATTTCAAAAGCATAGGAAAAATTTTTAGATGCCTAAGAATATACTCTTTACCTCCTGTACC

TCCCTACCAAATTTGTTCCTCTTTAAGTACCTGTTCCTGCTGAAAGCCGAGGAGACACCTGGATTGCCCTCTTTCTGTCCCTCCTGACCTCCAATGCATC

GGTTGGTTCATAAGCGGAAGAGCTGCTGCACTGTTCAGTATCATTTCCCCATATTTCACTGAGAAATGAAGTTCAGAGAGTAATGAAATTATGTAAGGGC

ATCTTTTTAAACAATACATTCAAGGACTTGGAATATAATTGGCAATTTTGTTGATGATGAATGAGGCTGAGCAATATAATGATGCCTTTTGCTACTCTAT

CCCTCTATTAAGACATAGGCTTACATGCATTCACTGAACAATGCTGGCTCCTGAAGAAAGCTTGGGAGTTTTGATAGAAGAGGATTTTAAGGTGTTTTCC

AACTGTTATATCACTTCCTAGTCCTGACCCTGGCCTTTTCTTAGACTTGTGATTTCCTAAAACTTGGGACAAGATGTTAAGGAAAAATATGTAGGCCCGC

CCCCTCCTGCTCTATCTAGGATTCATTCTTTTCTATTATTTGATTTTTTTTTTTGGCAGAAAAGTTCTTGTAGTATTGATATAAGTAAGAGCGAGAAAAA

TTGAGGGTATGAAGAGAAACACATTCCATGACATTTATTAAGTATGGTCTTGGCCATACTCTCTATCTTAGGGAAGCCCTGATGGAGGTGGTGGAATATG

ACTAGAGTAGTTTTGGTGATGCCCAAGATGGCAGCCTCACAGTGGTGGATTGGCCACATTTGGTTATACAAATGGCCAGTTTTCAATCAAAAACATTAGA

CTAAAGCAACAAAGAAGCAAGTTAAAAATTACACATTGCACTGACCATCTGACATGCAATGGCTTTGGGGACCAAGATGGATGTGGATAAGCTGAGAAAG

GCAGCTCACACTGGGGCTATATCTCTATGAGTGTTTTCCTAAAAACCTGAACACTCAGTATCTAATAGAGGTCAATAAGACTTCAATCCAGTTTCAGAGG

ACCAAGTACCTGTTATCTGTGAGGCACTATGCTAGAAAAAGAGATAACAACAGCTATCCTCCTGGAGTCTGTTATTTCAGTCAAGGTGATACAAATCAGC

AGAAGAGTGAGGTGTCTGGAATAAGAAATGAGGAAATTTTTTTTTTTTTTTTTTTTTGAGGCAGAGTCTTGCTCTGTCACCCAGGCTGGAGTGCAGTGGC

GAATGGTAGGCCATAAGAAGACAGGACAGTCAGAACCCAAATCAGCAATTTTGTCAGCACAAACAGCAACAGTAACAAGAAATGTTGACTGAAGCAACTG
