# Supplementary figures and images for: Sequence and Structure Characteristics of 22 Deletion Breakpoints in Intron 44 of the DMD Gene Based on Long-Read Sequencing
Source: Front Genet. 2021 Apr 30;12:638220. doi: 10.3389/fgene.2021.638220 (PMC8240811; doi:10.3389/fgene.2021.638220)

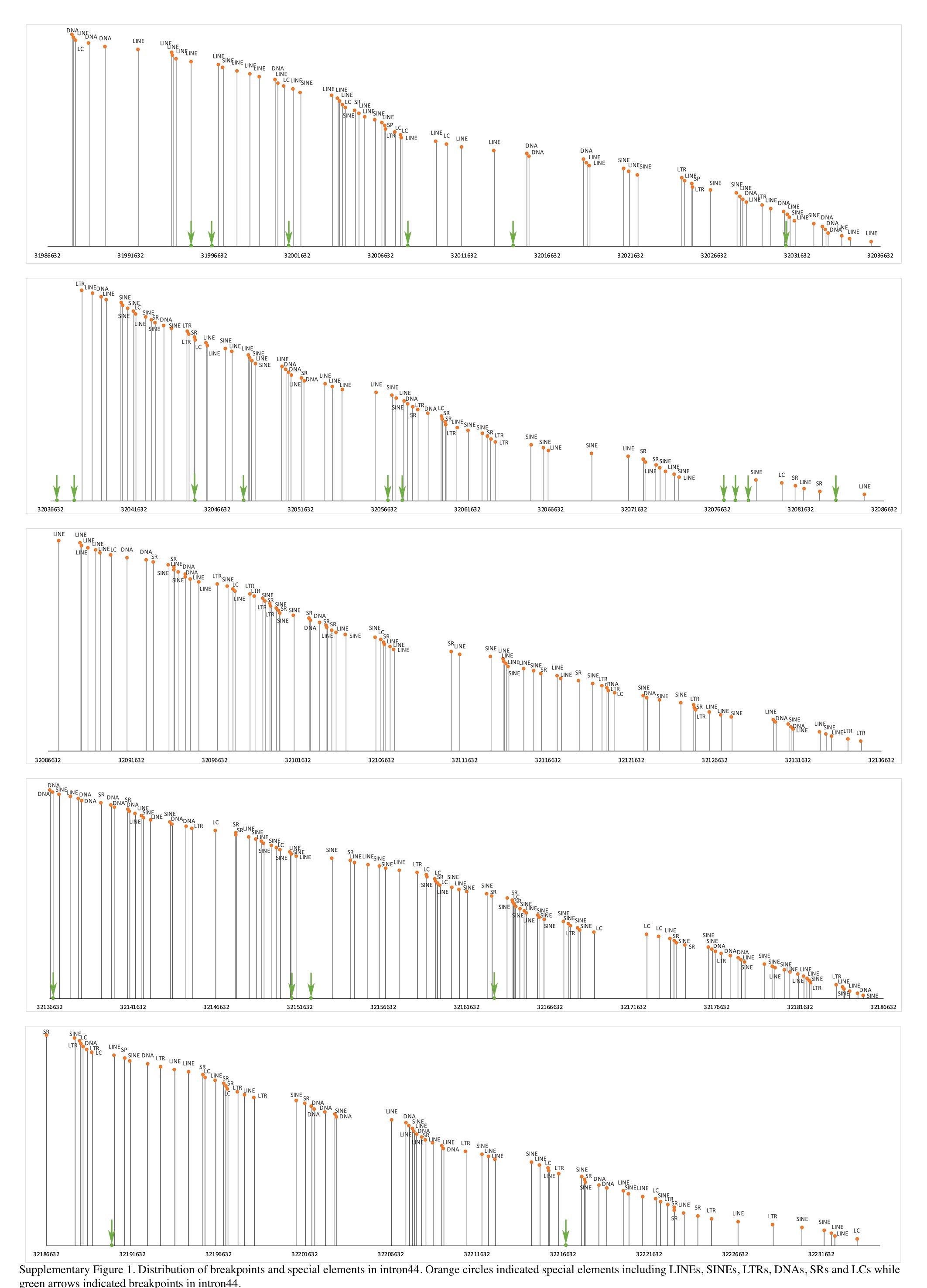

Supplement: Supplementary file 5 [file Image_1.jpeg]
